# The Influence of Medium Composition on EUCAST and Etest Antifungal Susceptibility Testing

**DOI:** 10.3390/jof9100973

**Published:** 2023-09-27

**Authors:** Roya Vahedi-Shahandashti, Melanie Maria Stubenböck, Cornelia Lass-Flörl

**Affiliations:** Institute of Hygiene and Medical Microbiology, Medical University of Innsbruck, 6020 Innsbruck, Austria; roya.vahedi@i-med.ac.at (R.V.-S.);

**Keywords:** CLSI, antifungal susceptibility testing (AFST), defined medium, complex medium, tested medium, RPMI 1640, *Candida*, *Aspergillus*

## Abstract

There is an ongoing effort to optimize and revise antifungal susceptibility testing (AFST) methods due to the rising number of fungal infections and drug-resistant fungi. The rising antifungal resistance within *Candida* and *Aspergillus* species, which are common contributors to invasive fungal infections (IFIs), is a cause for concern, prompting an expanding integration of in vitro AFST to guide clinical decisions. To improve the relevance of in vitro AFST results to therapy outcomes, influential factors should be taken into account. The tested medium is one of several factors that could affect the results of AFST. The present study evaluated the effect of two complex media (Sabouraud dextrose and Columbia) versus the standard defined medium (RPMI 1640) on the AFST results of amphotericin B, posaconazole, and voriconazole against *Candida* spp. and *Aspergillus* spp. representatives, utilizing the European Committee on Antimicrobial Susceptibility Testing (EUCAST) and the Etest methods. Overall, *Candida* species exhibited higher variability in minimum inhibitory concentration (MIC) across different media (more than three log2 dilutions) comparing to *Aspergillus* spp., while quality control isolates showed consistency regardless of tested media, antifungals, and methods. When comparing tested methods, MIC variation was mostly detected using EUCAST than it was using Etest.

## 1. Introduction

Over the past decade, fungal infections have become a growing threat to human health [1]. Invasive fungal infections (IFIs) are associated with significant morbidity and mortality risk, particularly for immunocompromised individuals, further worsened by the need for appropriate diagnostic approaches and therapeutic strategies [1,2]. IFIs have become even more problematic in recent years as several changes occurred, including an increase in patients at risk such as those with profound immunosuppression and an alarming rise in antifungal resistance, namely in *Aspergillus* and *Candida* species [3,4]. Considering the constant change in the IFIs epidemiology [5] and increasing choice in the antifungal armamentarium [6,7], it is more imperative than before to obtain accurate data on antifungal susceptibility testing (AFST) in vitro for optimizing treatment and predicting clinical outcomes.

The purpose of an AFST is to assist in choosing the most effective antifungal agent and thereby predict whether fungal infections will or will not respond to treatment [8,9]. To differentiate between susceptibility and resistance in vitro, AFST determines the minimum inhibitory concentration (MIC) required to inhibit an organism to a specified degree [10]. To reliably predict outcomes based on AFST results, reproducible techniques are crucial. Many factors can influence the results of in vitro susceptibility testing, including composition of the growth medium, temperature and time of incubation, concentration and method of preparation of the inoculum, and method of visual end-point determination [8,11]. Therefore, standardization by the Clinical and Laboratory Standards Institute (CLSI) [12] and the European Committee on Antimicrobial Susceptibility Testing (EUCAST) [13] was intended to minimize the impact of factors mentioned above on final MIC values, create uniformity in reporting, and facilitate interlaboratory comparisons [8].

Despite the standardized methods described by CLSI and EUCAST, both organizations periodically revise their guidelines to reflect the most current scientific research and clinical developments. There are several possible reasons for interlaboratory variation in antifungal MIC data, and one of the crucial factors is the medium composition [14,15,16]. It is unclear what the best nutrient medium is, as there is no consensus regarding the optimal nutrient medium. In theory, a nutrient medium should facilitate adequate fungus growth without interfering with the antifungal agent’s activity and produce reproducible results [9,17]. The RPMI 1640 medium, chosen as the standard medium for AFST by EUCAST and CLSI, has been shown to provide reproducible results, namely for yeast; however, this is not always the case, and there is an ongoing debate about its suitability and effectiveness [11,17]. It is important to note that isolates can be classified as either susceptible or resistant depending on the culture medium used for the assay. Therefore, it is crucial to carefully consider the selection of culture medium, as it can greatly affect the results of susceptibility testing. In order to address these concerns, the present study prospectively evaluated the effect of two reach media widely used in routine clinical laboratories (Sabouraud dextrose and Columbia) compared with the standard medium of EUCAST and CLSI guidelines (RPMI 1640) on some representative species of *Aspergillus* and *Candida*, two of the most common causative agents of fungal infections.

## 2. Material and Methods

### 2.1. Fungal Isolates and Identification

In total, twelve clinical isolates of *Candida* species (including *C. albicans* (*n* = 2), *C. glabrata* (*n* = 2), and *C. parapsilosis* (*n* = 2)) and *Aspergillus* species (including *A. fumigatus* (*n* = 2), *A. flavus* (*n* = 2), and *A. terreus* (*n* = 2)) were examined in this study. Species were identified by using matrix-assisted laser desorption ionization–time-of-flight mass spectrometry (MALDI-TOF MS) analysis, as previously described [18]. Some of the tested isolates were resistant to antifungals based on the AFST results, as follows: *C. albicans* 1 (voriconazole-resistant), *C. parapsilosis* 2 (voriconazole-resistant), and *A. fumigatus* 1 and 2 (voriconazole-resistant). Quality control (QC) isolates of *C. parapsilosis* (ATCC 22019), *C. krusei* (ATCC 6258), *A. fumigatus* (ATCC 204305), and *A. flavus* (ATCC 204304) were included every time testing was conducted.

### 2.2. Antifungal Agents and Susceptibility Testing

For broth microdilution AFST, antifungal powders of amphotericin B (AmB) (Sigma-Aldrich, Vienna, Austria, A2411) (solvent; dimethyl sulfoxide, Sigma-Aldrich, Vienna, Austria), voriconazol (VRC) (Sigma-Aldrich, Vienna, Austria, 0000216962) (solvent; dimethyl sulfoxide, Sigma-Aldrich, Vienna, Austria), and posaconazole (PSC) (Sigma-Aldrich, Vienna, Austria, 0000140447) (solvent; dimethyl sulfoxide, Sigma-Aldrich, Vienna, Austria) were utilized. For Etest AFST, commercialized gradient strips for AmB (0.002–32 mg/L; BioMérieux, Vienna, Austria, 1009558610), VRC (0.002–32 mg/L; BioMérieux, Vienna, Austria, 1009462370), and PSC (0.002–32 mg/L; BioMérieux, Vienna, Austria, 1009243550) were applied.

Isolates were cultured from 10% glycerol frozen stocks (−80 °C) on Sabouraud dextrose agar (SDA) (BD, Difco™, Le Pont de Claix, France) at 37 °C for 24 h for yeasts and up to 3 days for molds. Broth microdilution AFST was carried out according to “Eucast Definitive Document E.Def 9.3.2” for molds and “Eucast Definitive Document E.Def 7.3.2” for yeasts, using the following media: RPMI 1640 (Sigma-Aldrich, Vienna, Austria, R6504) supplemented with 2% glucose (Carl Roth, Karlsruhe, Germany) buffered to pH 7.0 with 0.165 M morpholinepropanesulfonic acid (MOPS) (Carl Roth, Karlsruhe, Germany), Sabouraud dextrose broth (SDB) (Millipore, Darmstadt, Germany), and Columbia broth (CB) (BD, Difco™, Le Pont de Claix, France). As previously described [19], the Etest AFST was performed according to manufacturer instructions, using the following media: RPMI 1640 agar, Sabouraud agar (SDA) (Biomérieux, Le Pont de Claix, France), and Columbia agar (CA) with 5% sheep blood (BD BBL™, Le Pont de Claix, France). At least three replicates of all experiments were performed.

### 2.3. Interpretation of Results

A final reading of MIC results of EUCAST for molds was performed with an inverted magnifying mirror after 48 h as the lowest drug concentration with complete inhibition of growth for all the tested antifungals. For yeasts, MIC results were obtained by a microtiter plate reader (TECAN, Sunrise™ absorbance reader, 30190079, Vienna, Austria) (530 nm) as follows: for AmB, the lowest concentration with ≥90% inhibition, and for azoles, the lowest concentration with ≥50% inhibition, compared to the drug-free control. The Etest MIC was the lowest drug concentration where the edge of the elliptical inhibition reached the antifungal strip’s scale. No consideration was given to microcolonies inside the elliptical zone. A MIC difference greater than three log2 dilutions was considered a variation among media.

## 3. Results

Susceptibility profiles of AmB, VRC, and PSC performed in three different media (RPMI, SDA, and CA) according to the EUCAST and Etest methods of *Candida* species are displayed in Table 1 and Table 2, respectively.

None of the tested *Candida* species showed variations in MICs of AmB by EUCAST and Etest methods (Table 1 and Table 2). In contrast, both representative isolates of *C. albicans* tested by the EUCAST method in SDB and CB showed higher VRC MIC than in RPMI 1640 medium (Table 1). A similar pattern was observed in one of the *C. albicans* isolates (no. 1) regarding the PSC MIC, which was higher in SDB and CB but not in RPMI 1640. In contrast, the second *C. albicans* isolate (no. 2) showed a higher PSC MIC in CB, but not in SDB or RPMI 1640 media. Additionally, the VRC and PSC MICs of *C. glabrata* no. 1 in SDB were higher than in RPMI 1640 and CB (Table 1). Quality control isolates appeared consistent regardless of testing media, methods, and species. According to the Etest results of azoles, only one of the *C. glabrata* isolates (no. 2) showed variation in VRC MIC on CA as compared to SDA and RPMI 1640 agar.

Neither EUCAST nor Etest detected significant MIC variations for any antifungal agent tested in different media against *Aspergillus* species (Table 3 and Table 4) except for *A. terreus* isolates, which had a higher AmB MIC in SDB and CB compared to RPMI 1640 by EUCAST. In general, *Candida* species showed greater MIC variations in different media (more than 3 log2 dilutions) than *Aspergillus* species, while quality control isolates showed consistency.

## 4. Discussion

There is an ongoing effort to optimize and standardize AFST methods due to the increasing number of fungal infections and the emergence of drug-resistant fungi [1,20]. However, susceptibility testing is not a guaranteed answer to questions about fungal diseases treatment, and other factors, including both patient and drug characteristics, also affect aspects that determine response to therapy; however the detection of antifungal resistance provides practical information to the clinician regarding the appropriateness of therapy [8,21]. A reproducible AFST technique is crucial for partially predicting the outcome of therapy. The results of in vitro susceptibility tests could be influenced by inoculum size, incubation time, temperature, shaking, and medium, revealing AFST’s numerous complexities [8,9,14]. Hence, the main rationale behind standardization and optimization by periodic revision of standard guidelines (CLSI and EUCAST) is to minimize the impact of such factors on the final MIC value.

Among the above-mentioned parameters, the medium used for in vitro susceptibility testing could be one of the most influential factors affecting an antifungal agent’s MIC value in several ways [22,23]. Isolates can be classified as either susceptible or resistant depending on the type of culture medium utilized for the assay. It is crucial to note that the selection of culture medium can have a significant impact on the outcome of susceptibility testing. Therefore, it is imperative to exercise caution and select the appropriate culture medium to obtain accurate test results. RPMI 1640 is the assay medium recommended by both CLSI and EUCAST guidelines, with slight modification in EUCAST (supplemented with 2% *w/v* glucose).

Fungal isolates may grow at varying rates on different media, resulting in diverse MIC values [17,24,25]. Due to MIC’s definition, which is the concentration of the drug that completely inhibits growth within a specified period, a sufficient amount of growth may not have occurred in certain media within that time, so the MIC value is therefore influenced by the organism’s slow growth rate rather than the drug’s inhibitory activity [8,17]. In addition, antifungal agents can also interact with medium component elements, and components of a particular medium may interfere with their uptake, altering the organism’s susceptibility [14]. It is, therefore, essential to select a suitable test medium that adequately supports fungal growth without drug interactions [26].

It has been shown in many studies that RPMI 1640 gives reproducible AFST results when tested for yeast [25,27]. The study conducted by Pfaller et al. [25] led to RPMI 1640 being chosen as a standard medium due to the high level of interlaboratory agreement reported [11]. However, the study also revealed that the suitability of RPMI 1640 was not significantly different from that of complex media such as the yeast nitrogen base (YNB) medium [25]. In addition, there was no evidence that this medium was suitable for filamentous fungi [17,22]. RPMI 1640 medium has two main advantages: it is a fully defined synthetic medium that is readily available commercially with minimal lot-to-lot variation, and it allows for the creation of an in vitro environment similar to what occurs in human body fluids during fungal infections since it is commonly used to culture mammals cells [14,27]. However, some drawbacks make RPMI 1640 a questionable medium as to whether it is appropriate for all fungi and antifungals, such as being a nutrient-limited medium affecting fungal growth [17] and, in some cases, not being able to discriminate amphotericin B-resistant and -tolerant isolates from susceptible ones [14,16,28,29].

Several studies showed that the susceptibility of yeast appears to be dependent on the fungus’s growth stage and the nutrient medium used, explaining how the medium might influence the MIC results [30,31]. Furthermore, the increased susceptibility of yeast in the exponential growth phase compared to the lag phase further highlights the significance of the medium in AFST [30]. Moreover, It was demonstrated that the susceptibility of filamentous fungi varied at different stages of the growth while cultured in the RPMI 1640 medium [32]. It was also shown how the type of the medium affects the interaction between antimicrobial agents and filamentous fungi [33]. Previous studies showed that complex undefined media, which are more nutritious, provide greater growth and appear to be associated with higher MICs than RPMI 1640, which does not support fungi growth [16,17]. Using a suboptimal medium like RPMI, which supports slow fungal growth, could lead to inaccurate MIC results. A fungus incapable of growing in a specific medium may falsely appear susceptible to an antifungal agent; however, in reality, the growth inhibition is caused by the medium itself rather than the antifungal agent.

The current study assessed the impact of two rich and undefined media, Sabouraud dextrose and Columbia, in comparison to RPMI 1640, which is considered a standard defined reference medium, on AFST results yielded by EUCAST and Etest methods. The objective was to investigate how the different tested media might influence the results of AFST. Overall, the present study found that tested *Candida* species showed more MIC variations in different media (more than 3 log2 dilutions) than *Aspergillus* species. The findings are consistent with a previous study that demonstrated that defined media like RPMI 1640 had the highest level of agreement in MIC of *Candida* species, while undefined media such as SDB showed more variation [25]. Interestingly, the increased MIC in the SDB and/or CB only has been detected in the tested *Candida* isolates and not in the quality control isolates (Table 1). The comparatively low MIC of *Candida* species in RPMI 1640 than CB and SDB could potentially explain the discrepancies between AFST results and therapy outcomes. Since quality control demonstrates good agreement among different media, it seems that RPMI 1640 is an appropriate medium for all other *Candida* species, while this is not always the case. As has been critically reviewed by de Sousa et al. [11], RPMI 1640 does not seem to be the most suitable growth medium of choice for all fungal species and antifungals, and as a consequence, advocating the use of a single preferred medium for susceptibility testing is problematic. Comparing the results yielded by EUCAST and Etest methods for *Candida* species separately, the most variation among tested media was found in EUCAST methods (Table 1 and Table 2), while Etest results seemed considerably less affected, which could be due to the difference in broth- and solid-based systems [19]. When considering *Aspergillus* species, the results were generally less affected than those for *Candida* species, regardless of the methods and media used. The only significant MIC variation was detected for the AmB MIC of *A. terreus* yielded by the EUCAST method, showing a higher MIC in complex media, SDB, and CB than RPMI 1640 (Table 3), which is consistent with our previous study [16]. Similar to *Candida* isolates, Etest results for *Aspergillus* species did not show significant differences among tested media (Table 4).

Considered as a whole, it was concluded that the concept that RPMI 1640 medium suits similarly all types of fungi and antifungals should be approached with caution. Although a broth microdilution technique employing RPMI 1640 medium shows consistent results for quality control strains, this does not necessarily imply that it is equally effective for all fungi and antifungals, mainly in the case of *Candida* species. The consistency resulting from RPMI 1640 medium, which poorly supports fungal growth, could be due to the growth inhibition caused by the medium rather than the antifungal agent. Therefore, consideration of growth control in each specific medium is an important requirement to differentiate the growth inhibition resulting from antifungals and from the medium.

## Figures and Tables

**Table 1:** The effect of medium on susceptibility tests of *Candida* isolates (*n* = 8) based on the EUCAST methods.

Species	Antifungals	MIC (mg/L)		
		RPMI 1640	SDB ^1^	CB ^2^
*C. albicans* (no. 1)	AmB	0.5	0.125	0.5
	VRC	0.03	≥4	≥4
	PSC	0.016	≥4	≥4
*C. albicans* (no. 2)	AmB	0.125	0.06	0.25
	VRC	1	≥4	≥4
	PSC	0.06	0.03	≥4
*C. parapsilosis* (no. 1)	AmB	0.25	0.5	0.5
	VRC	0.016	0.06	0.03
	PSC	0.03	0.008	0.008
*C. parapsilosis* (no. 2)	AmB	0.5	0.5	0.5
	VRC	1	4	0.5
	PSC	0.125	0.03	0.06
*C. glabrata* (no. 1)	AmB	0.25	0.06	0.25
	VRC	0.008	≥4	0.03
	PSC	0.016	≥4	0.008
*C. glabrata* (no. 2)	AmB	0.25	0.125	0.5
	VRC	2	4	1
	PSC	2	2	1
*C. parapsilosis* (ATCC 22019)	AmB	0.5	0.25	1
	VRC	0.016	0.125	0.06
	PSC	0.06	0.008	0.008
*C. krusei* (ATCC 6258)	AmB	0.5	0.5	0.5
	VRC	0.25	0.5	0.06
	PSC	0.06	0.03	0.03

AmB, amphotericin B; PSC, posaconazole; VRC, voriconazole. ^1^ SDB, Sabouraud dextrose broth. ^2^ CB, Columbia broth.

**Table 2:** The effect of medium on susceptibility tests of *Candida* isolates (*n* = 8) based on the Etest methods.

Species	Antifungals	MIC (mg/L)		
		RPMI 1640	SDA ^1^	CA ^2^
*C. albicans* (no. 1)	AmB	0.25	0.25	0.125–0.25
	VRC	0.06	0.25	0.016
	PSC	0.06	0.5	0.06
*C. albicans* (no. 2)	AmB	0.06	0.5	0.25
	VRC	2	4	1
	PSC	0.25	0.25	0.125
*C. parapsilosis* (no. 1)	AmB	0.03	0.25	0.125
	VRC	0.03	0.03	0.016
	PSC	0.03	0.03	0.06
*C. parapsilosis* (no. 2)	AmB	0.125	0.5	0.5
	VRC	1	1–2	1
	PSC	0.25	0.5	0.5
*C. glabrata* (no. 1)	AmB	0.06	0.25	0.25
	VRC	0.06	0.06	0.008
	PSC	0.016	0.03	0.03
*C. glabrata* (no. 2)	AmB	0.25	1	1
	VRC	4	4	>32
	PSC	>32	>32	>32
*C. parapsilosis* (ATCC 22019)	AmB	0.25	1	0.125
	VRC	0.06	0.06	0.03
	PSC	0.06	0.06	0.06
*C. krusei* (ATCC 6258)	AmB	0.5	1	1
	VRC	0.25	0.25	0.25
	PSC	0.25	0.125	0.25

AmB, amphotericin B; PSC, posaconazole; VRC, voriconazole. ^1^ SDA, Sabouraud dextrose agar. ^2^ CA, Columbia agar with 5% sheep blood.

**Table 3:** The effect of medium on susceptibility tests of *Aspergillus* isolates (*n* = 8) based on the EUCAST methods.

Species	Media	MIC (mg/L)		
		RPMI 1640	SDB ^1^	CB ^2^
*A. fumigatus* (no. 1)	AmB	0.5	2	1
	VRC	8	16	16
	PSC	2	2	2
*A. fumigatus* (no. 2)	AmB	0.5	2	1–2
	VRC	8	>16	16
	PSC	2	2	2
*A. flavus* (no. 1)	AmB	1	2	4
	VRC	1	2	2
	PSC	0.25	0.125	0.25
*A. flavus* (no. 2)	AmB	2	4	4
	VRC	1	2	2
	PSC	0.125	0.125	0.125
*A. terreus* (no. 1)	AmB	1	16	8
	VRC	2	8	8
	PSC	0.06	0.125	0.125
*A. terreus* (no. 2)	AmB	1	16	8
	VRC	1	2	2
	PSC	0.125	0.125	0.06
*A. fumigatus* (ATCC 204305)	AmB	0.5	2	1
	VRC	1	4	2
	PSC	0.125	0.25	0.125
*A. flavus* (ATCC 204304)	AmB	2	4	2
	VRC	1	2	2
	PSC	0.25	0.125	0.125

AmB, amphotericin B; PSC, posaconazole; VRC, voriconazole. ^1^ SDB, Sabouraud dextrose broth. ^2^ CB, Columbia broth.

**Table 4:** The effect of medium on susceptibility tests of *Aspergillus* isolates (*n* = 8) based on the Etest methods.

Species	Media	MIC (mg/L)		
		RPMI 1640	SDA ^1^	CA ^2^
*A. fumigatus* (no. 1)	AmB	0.125	0.5	0.5
	VRC	8	4	2
	PSC	2	2	2
*A. fumigatus* (no. 2)	AmB	0.125	0.5	0.5
	VRC	2	4	1
	PSC	1	2	0.5
*A. flavus* (no. 1)	AmB	0.5	2	1
	VRC	0.25	0.25	0.25
	PSC	0.25	0.25	0.25
*A. flavus* (no. 2)	AmB	2	4	4
	VRC	0.5	0.125	0.125
	PSC	0.125	0.06	0.125
*A. terreus* (no. 1)	AmB	0.25	1	2
	VRC	2	2	0.5
	PSC	0.125	0.125	0.125
*A. terreus* (no. 2)	AmB	0.25	2	2
	VRC	1	1	0.25
	PSC	0.125	0.25	0.125
*A. fumigatus* (ATCC 204305)	AmB	0.25	0.5	0.5
	VRC	0.125	0.25	0.125
	PSC	0.125	0.25	0.06
*A. flavus* (ATCC 204304)	AmB	1	2	2
	VRC	0.5	0.25	0.25
	PSC	0.125	0.125	0.125

AmB, amphotericin B; PSC, posaconazole; VRC, voriconazole. ^1^ SDA, Sabouraud dextrose agar. ^2^ CA, Columbia agar with 5% sheep blood.

## Data Availability

Not applicable.
